# Alterations of Regional Homogeneity in Preschool Boys With Autism Spectrum Disorders

**DOI:** 10.3389/fnins.2021.644543

**Published:** 2021-03-22

**Authors:** Zhihong Lan, Shoujun Xu, Yunfan Wu, Likun Xia, Kelei Hua, Meng Li, Mengchen Liu, Yi Yin, Chunlong Li, Shumei Huang, Ying Feng, Guihua Jiang, Tianyue Wang

**Affiliations:** ^1^Department of Medical Imaging, Guangdong Second Provincial General Hospital, Guangzhou, China; ^2^Department of Radiology, Shenzhen Children’s Hospital, Shenzhen, China; ^3^Department of Magnetic Resonance Imaging, People’s Hospital of Yuxi City, Yuxi, China

**Keywords:** autism spectrum disorders, regional homogeneity, resting-state fMRI, preschool boys, neural activity

## Abstract

**Objectives:**

The study was aimed at investigating the alterations of local spontaneous brain activity in preschool boys with autism spectrum disorders (ASD).

**Methods:**

Based on regional homogeneity (ReHo), the acquired resting state functional magnetic resonance imaging (fMRI) data sets, which included 86 boys with ASD and 54 typically developing (TD) boys, were used to detect regional brain activity. Pearson correlation analysis was used to study the relationship between abnormal ReHo value and the Childhood Autism Rating Scale (CARS), Autism Behavior Checklist (ABC), developmental quotient, and age.

**Results:**

In the ASD group, we found increased ReHo in the right calcarine as well as decreased ReHo in the opercular part of the left inferior frontal gyrus, the left middle temporal gyrus, the left angular gyrus, and the right medial orbital frontal cortex (*p* < 0.05, false discovery rate correction). We did not find a correlation between the results of brain regions and the CARS, ABC, and age.

**Conclusions:**

Our study found spontaneous activity changes in multiple brain regions, especially the visual and language-related areas of ASD, that may help to further understand the clinical characteristics of boys with ASD.

## Introduction

Autism spectrum disorders (ASD) have social disorders, communication difficulties, repetitive and stereotyped behaviors, and limited and narrow interests as the core symptoms. These neurodevelopmental disorders occur early in childhood. The latest large-scale global survey has estimated that the incidence rate of ASD is about 1–2%, and it has been steadily increasing (Lai et al., 2014). Although the neurobiological basis of ASD has been known for more than 40 years (Damasio and Maurer, 1978), the heterogeneity and the high proportion of co-occurring symptoms with other mental disorders (Lai et al., 2019), the independent brain basis of ASD, has not been clearly identified in individuals.

In recent years, the development of neuroimaging provides a new platform for the study of the neural changes of ASD. Dajani and Uddin (2016) divided their subjects into three groups—children, adolescents, and adults—for Regional homogeneity (ReHo) analysis. The authors found that, compared with the typically developing (TD) group, all three groups of patients with ASD had abnormal spontaneous neural activity, which changed with age, especially among the children. ReHo is a method that uses Kendall’s coefficient of concordance (KCC) to measure the similarity of the time series of a given voxel with its 26 nearest neighbors in the brain. ReHo, which can reflect the strength of local spontaneous neural activity in the brain, was used in the study of ASD (Paakki et al., 2010; Shukla et al., 2010; Itahashi et al., 2015; Li et al., 2018), but the results were heterogeneous. Jao Keehn et al. (2019) also used the ReHo analysis and found that there was spontaneous nerve activity enhancement in the pericalcarine visual cortex in adolescent patients with ASD (8–18 years old). In another ASD study of unlimited age groups, it was found that, in the ASD group, the local spontaneous nerve activity in the occipital and posterior temporal regions was increased, and that in the middle/posterior cingulate gyrus and medial prefrontal lobe was decreased (Maximo et al., 2013). The researchers believed that the heterogeneity in the current results may be attributed to the different age of patients with ASD.

Autism spectrum disorders has always been considered to be a disease characterized by early changes in brain development, as early as 6–12 months of age, where there is excessive growth of the cortical surface area (Hazlett et al., 2017). With the increase of age and treatment intervention, patients with ASD may have compensatory neural functional activity (Mevel et al., 2015). Furthermore, they are more prone to co-occurring insomnia or other mental disorders (Lai et al., 2019). All these factors may lead to heterogeneity in the brain functional changes of ASD patients at different ages. In consequence, narrowing the age span of patients with ASD in younger children seems likely to produce purer, more reliable results. However, there have been few reports of resting-state functional magnetic resonance imaging (fMRI) studies based on ReHo of ASD patients under 7 years old.

In addition, more studies have begun to focus on sex differences in ASD. Although the morbidity of ASD is two to five times more common in men than in women (Ecker et al., 2017), sex differences in ASD (including clinical and genetic/neurobiological aspects) have gone beyond the scope of epidemiological studies (Lai et al., 2015). For example, in clinical manifestations, males exhibit more restrictive, repetitive, and stereotyped behaviors than females although females exhibit more comorbid psychopathology (Retico et al., 2016). Brain structural imaging studies also found differences in neuroanatomic regions (e.g., corpus callosum volume) among ASD patients of different sexes (Lai et al., 2017). However, in previous fMRI studies of ASD, sex was rarely separated.

Therefore, our study intended to acquire resting-state fMRI data based on ReHo analysis from a relatively large sample size of preschool boys (3–6 years old) to specifically explore the alterations of local spontaneous activity in younger children with ASD and analyze its correlation with the severity of symptoms in patients with ASD. Based on a literature review, we speculated that there might be abnormal spontaneous neural activity in the visual cortex and semantic system–related brain areas in young children with ASD.

## Subjects and Methods

### Subjects

We recruited 86 ASD preschool boys and 54 age-matched TD boys from Shenzhen Children’s Hospital. Healthy boys were recruited into the TD group through advertisement. All data sets were collected from November 2016 to August 2018. The study was approved by the ethics committee of Shenzhen Children’s Hospital. The guardian of each subject was asked to sign informed consent forms on the premise of fully understanding the purpose of the study. Before the scans, all children with ASD and TD who met the following criteria were excluded: neurological disorders (e.g., epilepsy and Tourette’s syndrome), genetic disorders (e.g., Fragile X and Rett syndrome), or psychiatric disorders (e.g., childhood disintegrative disorder, selective mutism, obsessive-compulsive disorder, or Asperger’s syndrome). Children with a history of severe physical illness, a history of loss of consciousness for more than 5 min, or who were currently taking psychoactive drugs were also excluded.

### Methods

#### Clinical Assessment

All ASD subjects were co-diagnosed by two associate chief physicians of pediatrics and psychiatry, met the ASD DSM-V criteria and cutoffs on all the Childhood Autism Rating Scale (CARS) (Schopler et al., 1980) and Autism Behavior Checklist (ABC) (Krug et al., 1980) domains. CARS and ABC are the main diagnostic and screening tools for ASD in Chinese children. CARS, which is suitable for people over 2 years old, was assessed by a trained doctor. Although the diagnostic sensitivity of CARS for ASD is not as high as the Autism Diagnostic Observation Schedule, which is known as the gold standard, the CARS has a higher specificity (Randall et al., 2018), which can avoid the inclusion of over-diagnosed children in the ASD group. The ABC was completed by parents of the subjects and is applicable to persons aged 8 months to 28 years. We also used the developmental diagnostic scale, which is suitable for children aged 0–6 years to assess the developmental quotient (DQ) (DQ < 70 as a low score).

#### MRI Data Acquisition

Image data acquisition was performed by two experienced radiographers. Each child received 0.5% chloral hydrate 0.5 ml/kg (maximum dose 10 ml) and was sedated by a certified nurse. During the entire scan, each participant was required to have a caregiver and guardian present.

MRI data were collected by a 3T Siemens Skyra scanner from the radiology department of Shenzhen Children’s hospital. Each participant was in the supine position, and the head was fixed firmly on a foam pad to prevent head movement. Adhesive earmuffs were also used to plug the ears to protect hearing. Then the resting-state fMRI data set was obtained using the gradient echo-planar imaging sequence. Resting-state fMRI collection parameters were as follows: repeat time (TR)/echo time (TE): 2000 ms/30 ms; flip angle: 90°; thickness/interval: 3.6 mm/0.72 mm; field of view (FOV): 230 × 230 mm; matrix: 64 × 64, and layers: 35. In 8 min, 240 volumes were obtained. After the MRI scan, images of each participant were examined to ensure that they met the experimental requirements. Meanwhile, a T1-weighted sequence of magnetization-prepared rapid-acquisition gradient echo (MPRAGE) prepared by three-dimensional magnetization covering the whole brain (176 sagittal sections) was obtained. The corresponding acquisition parameters were set as TR: 2300 ms, TE: 2.26 ms, TI: 900 ms, flip angle: 8°, acquisition matrix: 256 × 256, FOV: 256 × 256 mm, and layer thickness was 1 mm.

#### Data Processing and ReHo Calculations

Image preprocessing was performed using the data processing assistant in the resting state fMRI toolbox (DPARSF 3.0 Advanced Edition,^1^). For each participant, the first 10 time points were discarded due to transient signal changes before magnetization reached a stable state and the participant adapted to the fMRI noise. To minimize the influence of head movement, subjects with maximum displacement greater than 1.5 mm and angle movement greater than 1.5° during the whole fMRI scan were excluded. No subjects were deleted in this step. Then, the resting-state fMRI data were corrected for intra-volume acquisition delay and co-registered together with anatomical scanning. The co-registered anatomical images were divided into gray matter, white matter, and cerebrospinal fluid. Then, all data spaces were normalized to the whole brain template of the Montreal Neurological Institute standard space (age 4.5–8.5 years) with an isotropic voxel size 3 mm × 3 mm × 3 mm (Fonov et al., 2011). Furthermore, a regression analysis was conducted to minimize the influence on head motion (Friston 24 model), cerebrospinal fluid, and white matter, following which the high-frequency physiological noise and low-frequency drift were filtered (0.01–0.08 Hz).

The ReHo calculation process was the same as that reported in previous studies (Zang et al., 2004). In short, ReHo was estimated on a voxel-by-voxel basis by calculating the KCC of a given voxel time series with its nearest 26 adjacent voxel time series. The KCC value was calculated as the voxel, and a separate KCC map was obtained for each subject. The data were then spatially smoothed with an 8-mm full-width at half-maximum Gaussian kernel to reduce the noise and residual in the gyrus anatomy.

#### Statistical Analysis

Two independent-sample, nonparametric tests were used to assess the age difference between ASD boys and TDs. To explore the differences in ReHo between ASD boys and TDs, the REST-State fMRI Data Analysis Toolkit (REST 1.8) was used to conduct a two-sample *t*-test on a single, normalized ReHo map, and the brain regions that had significant differences were found after the false discovery rate correction with *p* < 0.05. To determine the relationship between the ReHo alteration in different brain regions and the CARS, ABC, DQ, and age, Pearson correlation analysis was conducted in SPSS 20.0 software (*p* < 0.05).

## Results

### Demographic and Clinical Characteristics

All of the data of the ASD and TD groups were retained after processing. There was no significant difference in age between the two groups. The values of the ABC, CARS, and DQ of the ASD group were consistent with the ASD standard as shown in Table 1. TD subjects were not measured by the corresponding scale.

**TABLE 1:** Demographic and clinical characteristics of ASD boys and TDs.

	ASD group (*n* = 86)	TD group (*n* = 54)	*Z*	*P*
Age	3.92 ± 0.95	4.09 ± 0.96	−1.693	0.09
ABC	68.12 ± 15.15			
CARS	34.17 ± 2.08			
DQ	53.44 ± 7.90			

*Data are mean ± standard deviation. ABC, Autism Behavior Checklist; CARS, Childhood Autism Rating Scale; DQ, developmental quotient.*

### Regional Spontaneous Activity Changes

**FIGURE 1:**
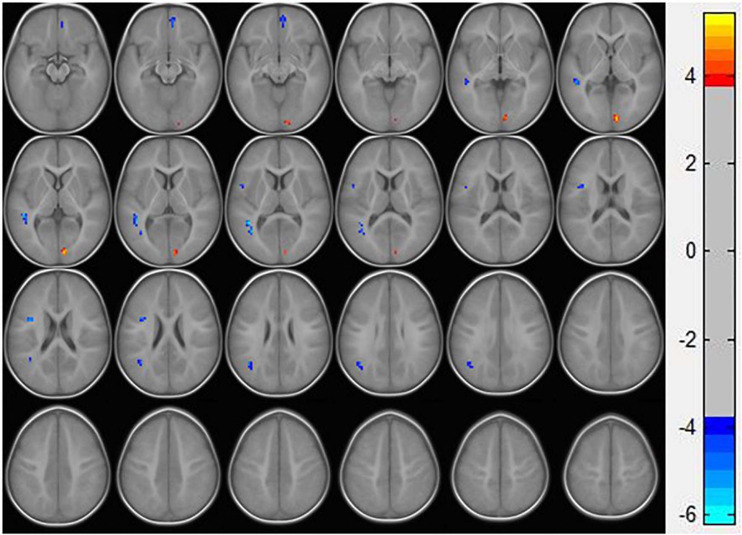
Statistically significant differences between ASD boys and TDs are shown in a ReHo map of the whole brain with MRI. The ASD boys showed a significant ReHo increase in the right calcarine (warm colors) as well as a decrease in the opercular part of the left inferior frontal gyrus, left middle temporal gyrus, left angular gyrus, and right medial orbital frontal cortex with decreased ReHo values (cold colors). A T-score bar is shown on the right. Warm and cold colors denote increases and decreases in ReHo, respectively.

**FIGURE 2:**
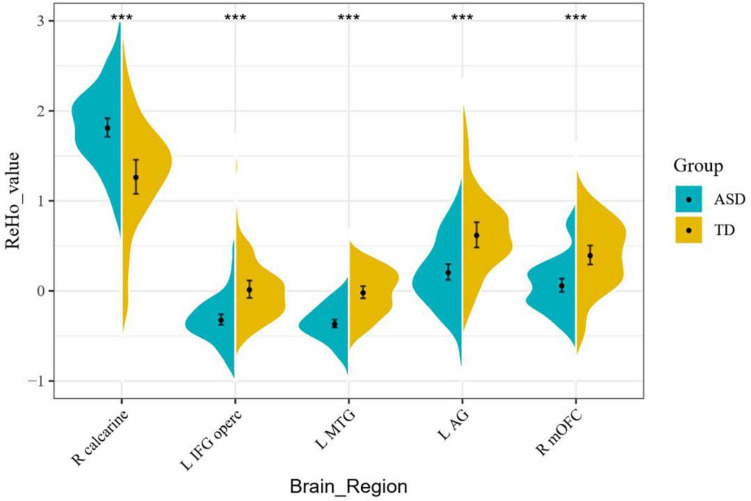
ReHo signal values for altered regional brain regions between ASD boys and TDs.

Resting-state fMRI analysis showed that, compared with the TD group, ASD boys had increased ReHo in the right calcarine as well as decreased ReHo in the opercular part of the left inferior frontal gyrus (IFG operc), left middle temporal gyrus (MTG), left angular gyrus (AG), and right medial orbital frontal cortex (mOFC) (Table 2 and Figures 1, 2).

**TABLE 2:** Brain regions with abnormal ReHo in ASD boys.

Brain region	Cluster size	MNI coordinates			AAL	Peak *T*-value
		*X*	*Y*	*Z*		
R calcarine	36	9	−90	3	Calcarine_R	5.1821
L IFG operc	22	−39	3	21	Frontal_Inf_Oper_L	−5.1215
L MTG	49	−42	−48	9	Temporal_Mid_L	−6.2140
L AG	27	−42	−57	27	Angular_L	−4.7905
R mOFC	21	6	51	−9	Frontal_Med_Orb_R	−4.8747

*L, left; R, right; MNI, Montreal Neurological Institute; ReHo, regional homogeneity. IFG operc, opercular part of inferior frontal gyrus; MTG, middle temporal gyrus; AG, angular gyrus; mOFC, medial orbital frontal cortex; AAL, anatomical automatic labeling.*

### Correlation Between ReHo Values in Abnormal Regions and CARS, ABC, DQ, and Age

The ReHo values of the five brain regions and the values of the ABC, CARS, DQ, and age were in accordance with normal distribution; hence, Pearson correlation analysis was used. However, we did not find a significant correlation between any of the five brain regions and ASD-related scales or age.

## Discussion

In this study, based on a large sample size of resting-state fMRI, we used ReHo analysis to explore the alterations of spontaneous neural activity in the brain of ASD preschool boys. The results show that the ReHo value in the right calcarine of the primary visual cortex was increased in preschool boys with ASD, and the ReHo value in the language-related brain region (left IFG operc, left MTG, and left AG) and right mOFC was decreased. No significant correlation was found between the abnormal brain regions and the score on the ASD scale.

We found that the ReHo value of the right calcarine increased, suggesting that there was an increase in local spontaneous neural activity in the right calcarine. The calcarine is an important part of the primary visual cortex, and abnormal visual processing in patients with ASD has been the focus of the research on a mechanism of ASD. Among them, many recent studies attributed the enhancement of the primary visual cortex of ASD patients to the functional compensation of semantic dysfunction (Samson et al., 2012; Chen et al., 2016); in fact, abnormal activity in the primary visual cortex of ASD patients at different ages has been frequently reported in previous studies. Peiker et al. (2015) found that the primary visual cortex of 24- to 45-year-old ASD patients had an abnormally strong neuromodulation to the intensity of visual stimuli by using magnetoencephalography. Based on ReHo analysis, Nair et al. (2018) also found local over-connectivity of the posterior visual region in the resting-state fMRI data of ASD patients aged 7–18 years. Similarly, the increase in spontaneous activity in the primary visual cortex was found in preschool-age boys with ASD in our study. Even before the onset of symptoms in ASD, 9-month-old children with a high risk of ASD who developed ASD subsequently also had an enhanced visual-search ability related to the severity of ASD that subsequently was identified (Gliga et al., 2015). In consequence, it is reasonable to assume that abnormal visual processing in patients with ASD is not the secondary result of possible functional compensation. In contrast to the compensatory viewpoint, using a dense-array electroencephalography method, Ronconi et al. (2018) found that individuals with ASD demonstrated an advantage in detail-focused tasks, which tended to suggest that the imbalance between neuroenhancement and inhibitory mechanisms in the primary visual cortex in patients with ASD contributes to this phenomenon. Thus, we suggest that increased spontaneous activity of the primary visual cortex may be one correlative variable of ASD itself.

Simultaneously, we found that there was a decrease of spontaneous neural activity in the left IFG operc and left MTG in preschool boys with ASD. The former is a part of Broca’s area, which is an important area related to semantic expression and comprehension (Bednarz et al., 2017), whereas the latter is an important part of the semantic system in Wernicke’s brain area, which is mainly responsible for semantic representation and vocabulary storage (Binder et al., 2009). The left MTG can assist the left IFG in semantic understanding and retrieval from vocabulary storage to participate in the selection process of lexical association degree (Wong et al., 2019). Previous studies have found that, in ASD patients, the left MTG and left IFG present simultaneous decrease activation (Ogawa et al., 2019) and reduce connectivity between them (Sahyoun et al., 2010). Moreover, with the improvement of language comprehension in ASD patients, after reading intervention, the reduced connectivity between the left MTG and the left IFG can be restored (Murdaugh et al., 2016). The results that we found showed a decrease in spontaneous neural activity in the left IFG operc and left MTG, which is consistent with previous studies. Therefore, although the decrease of spontaneous neural activity in the left IFG operc and left MTG we found has no significant correlation with the score on CARS and ABC, we still have reason to speculate that these decreases are closely related to the early language development delay of ASD.

In this study, decreased local spontaneous neural activity in the left AG was also found. The AG is considered an important brain region responsible for episodic memory. The AG may play a particular role in behaviors requiring fluent conceptual combination, such as sentence comprehension, discourse, problem solving, and planning (Binder et al., 2009). Some researchers have found that inhibiting AG activity can reduce subjects’ retrieval of episodic details in past and future events (such as time, place, and person), thus interrupting or weakening episodic simulation and memory (Thakral et al., 2017). Thus, we speculate that the decreased local spontaneous neural activity of the left AG may be related to the symptom of episodic prediction and episodic memory as well as the inversion of words and poor intelligibility in language expression of ASD children. In addition, the abnormal AG was also found in previous studies of ASD magnetoencephalography (Lajiness-O’Neill et al., 2018), and the impairment of episodic memory in ASD patients has been repeatedly replicated (Hutchins and Prelock, 2018), which further demonstrates our view.

The decreased local spontaneous neural activity in the right mOFC was observed in this study as well. The mOFC, as a part of the prefrontal cortex, is thought to be associated with complex emotions and reward and punishment processing, which are essential for social behavior (Kandilarova et al., 2019). Based on fMRI analysis of patients with obsessive-compulsive disorder (OCD), Fan et al. (2017) found that the abnormality of the right mOFC may cause patients with OCD to lose the ability to judge the value of compulsive behaviors, resulting in compulsive behaviors. Another fMRI neuro-basic study of ASD in adolescents also found that reduced OFC activation was associated with abnormal reward decision making in patients with ASD (Carlisi et al., 2017). We replicated this result, which may indicate that ASD patients have altered their reward decision making since childhood, resulting in a failure to make value judgments about repetitive and stereotyped behaviors. This is also consistent with the finding that males with ASD exhibit more stereotypical behaviors than females (Retico et al., 2016).

We did not find a correlation between the ASD preschool boys’ abnormal brain regions and the ASD score scale. Possible reasons may be that (Lai et al., 2014) our study did not further subdivide the ASD group into high- or low-functioning autism or (Damasio and Maurer, 1978) the boys with ASD in our study were too young to score by themselves, so the CARS (scored by a doctor) and ABC (scored by a parent) are subjective. Therefore, we still cannot rule out that these abnormal brain regions do correlate with the severity of ASD.

There are some limitations in our study. First, we only investigated the ReHo of preschool boys with ASD although ASD has atypical brain development trajectories (Mevel et al., 2015) and sex differences (Ecker et al., 2017). As such, the interpretation of our results cannot be extended to ASD boys of other ages or females. Second, the results we observed still cannot completely explain that secondary changes in brain development of ASD have been excluded. In fact, 9-month-old high-risk ASD infants (later diagnosed with ASD) have been shown to have enhanced visual search ability (Cheung et al., 2018). Therefore, our next research analysis should focus on the prospective study of brain function alterations in high-risk ASD children.

Finally, based on resting-state fMRI data, the brain regions with functional abnormalities in preschool boys with ASD were found by using ReHo analysis. We suggest that increased spontaneous activity of the primary visual cortex might be a potential primary disorder of boys with ASD. In addition, some brain regions (e.g., the left IFG operc, left MTG, and left AG and right mOFC) may partly explain the core symptoms of ASD, such as social dysfunction and stereotyped behavior(s). Given that more than 60% of children with ASD are prescribed for any clinical indication and that more than 41% of them are taking more than one psychotropic drug with limited effect (Lamy et al., 2020), these results may help us to provide new targets for treatment and better understand the clinical features of preschool boys with ASD.

## Data Availability Statement

The raw data supporting the conclusions of this article will be made available by the authors, without undue reservation.

## Ethics Statement

The studies involving human participants were reviewed and approved by the Ethics Committee of Shenzhen Children’s Hospital. Written informed consent to participate in this study was provided by the participants’ legal guardian/next of kin.

## Author Contributions

ZL: validation, formal analysis, investigation, writing – original draft, and visualization. SX: software, investigation, resources, and funding acquisition. YW: methodology and software. LX, KH, and McL: methodology. ML and YY: software. CL, SH, and YF: validation. GJ: conceptualization, data curation, writing – review and editing, supervision, project administration, and funding acquisition. TW: formal analysis, data curation, writing – review and editing, and funding acquisition. All authors contributed to the article and approved the submitted version.

## Conflict of Interest

The authors declare that the research was conducted in the absence of any commercial or financial relationships that could be construed as a potential conflict of interest.

## Footnotes

1 http://rfmri.org/DPARSF

## Abbreviations

ASD: autism spectrum disorders
ReHo: regional homogeneity
fMRI: functional magnetic resonance imaging
TD: typically developing
CARS: childhood autism rating scale
ABC: autism behavior checklist
DQ: developmental quotient
IFG operc: the opercular part of inferior frontal gyrus
MTG: middle temporal gyrus
AG: angular gyrus
mOFC: medial orbital frontal cortex
FDR: false discovery rate
KCC: kendall’s coefficient of concordance
ADOS: autism diagnostic observation schedule
FOV: field-of-view.
